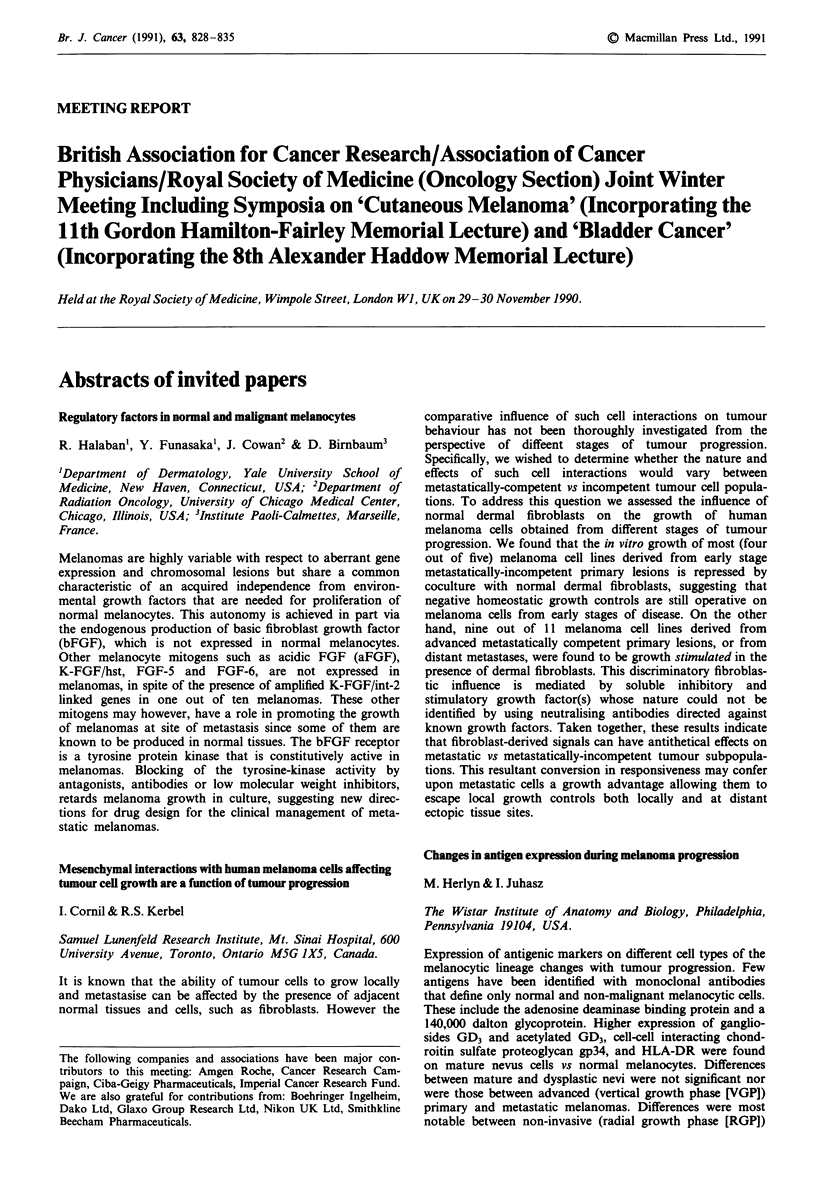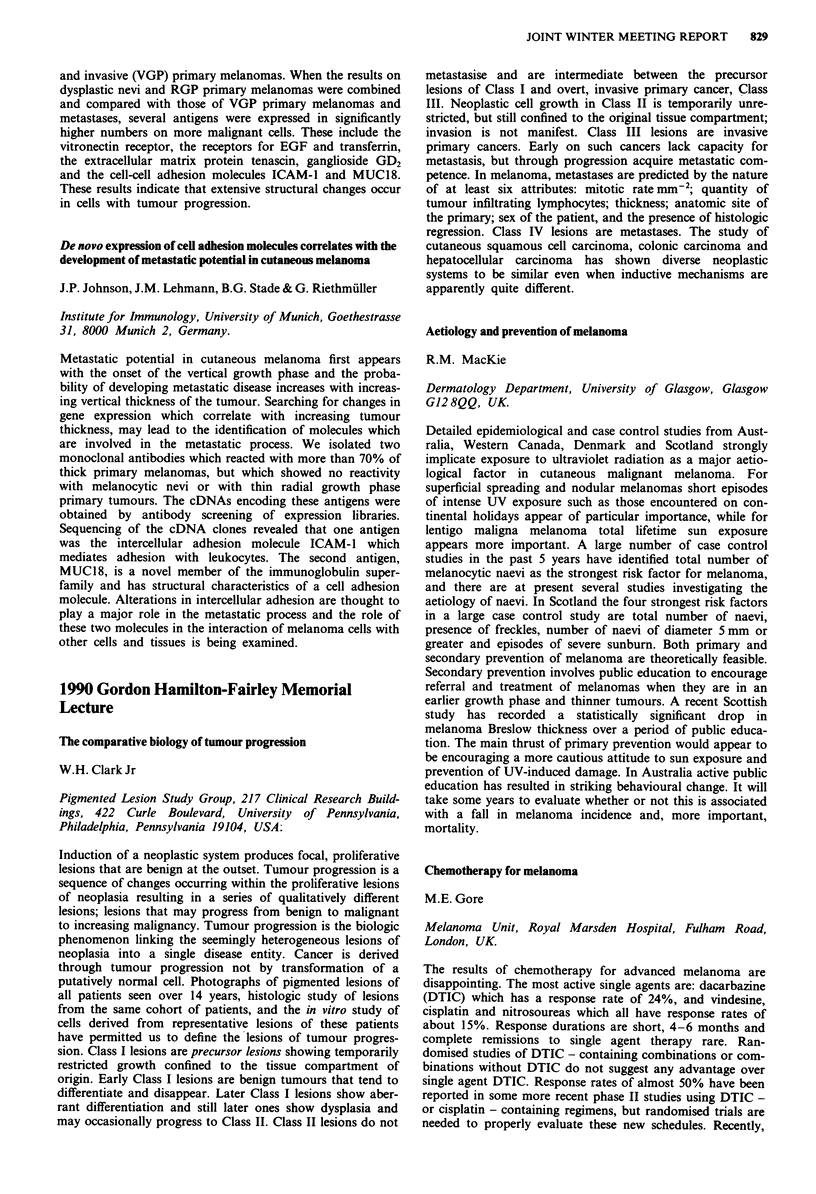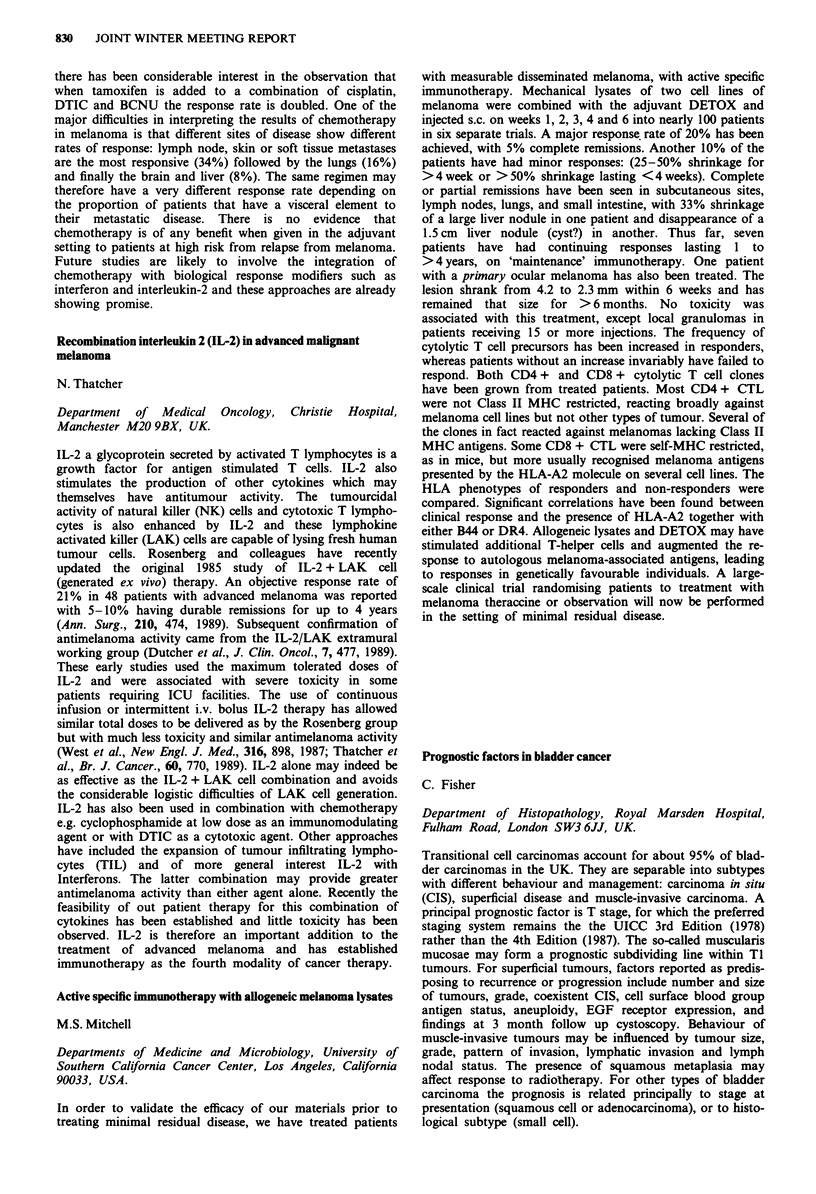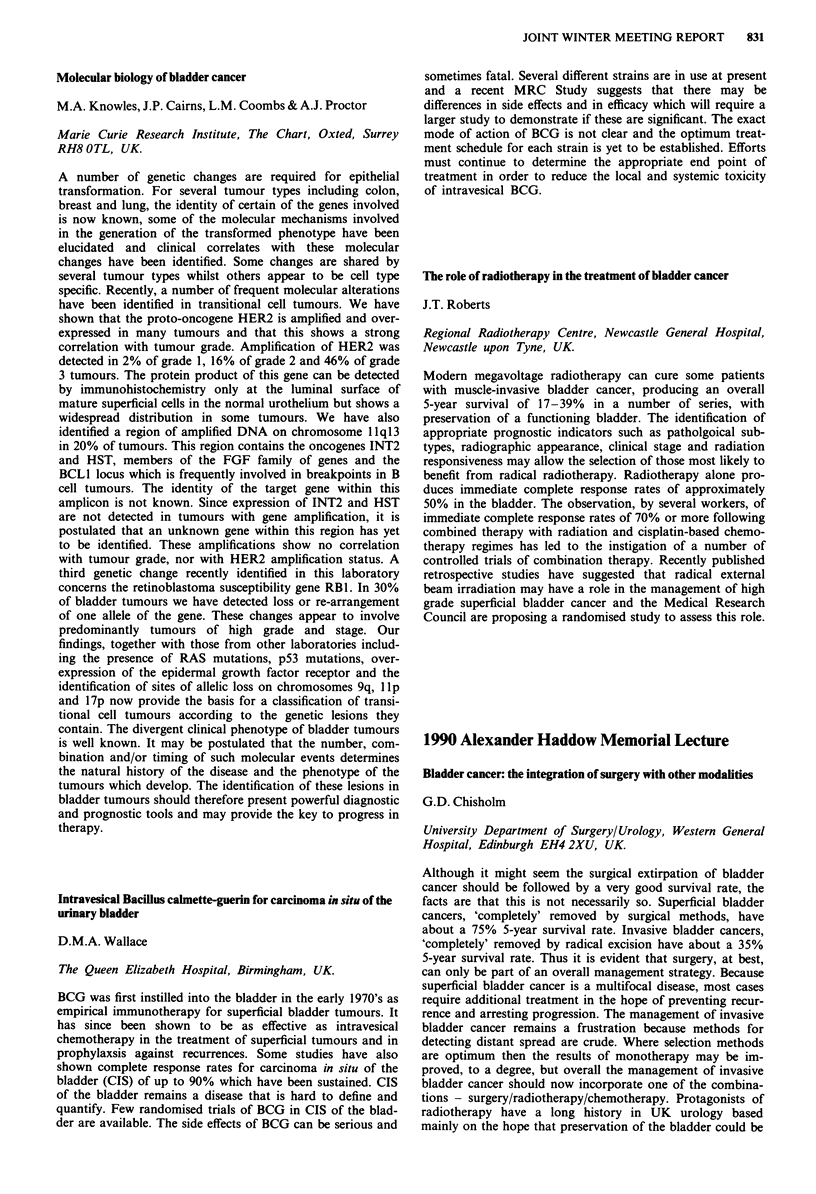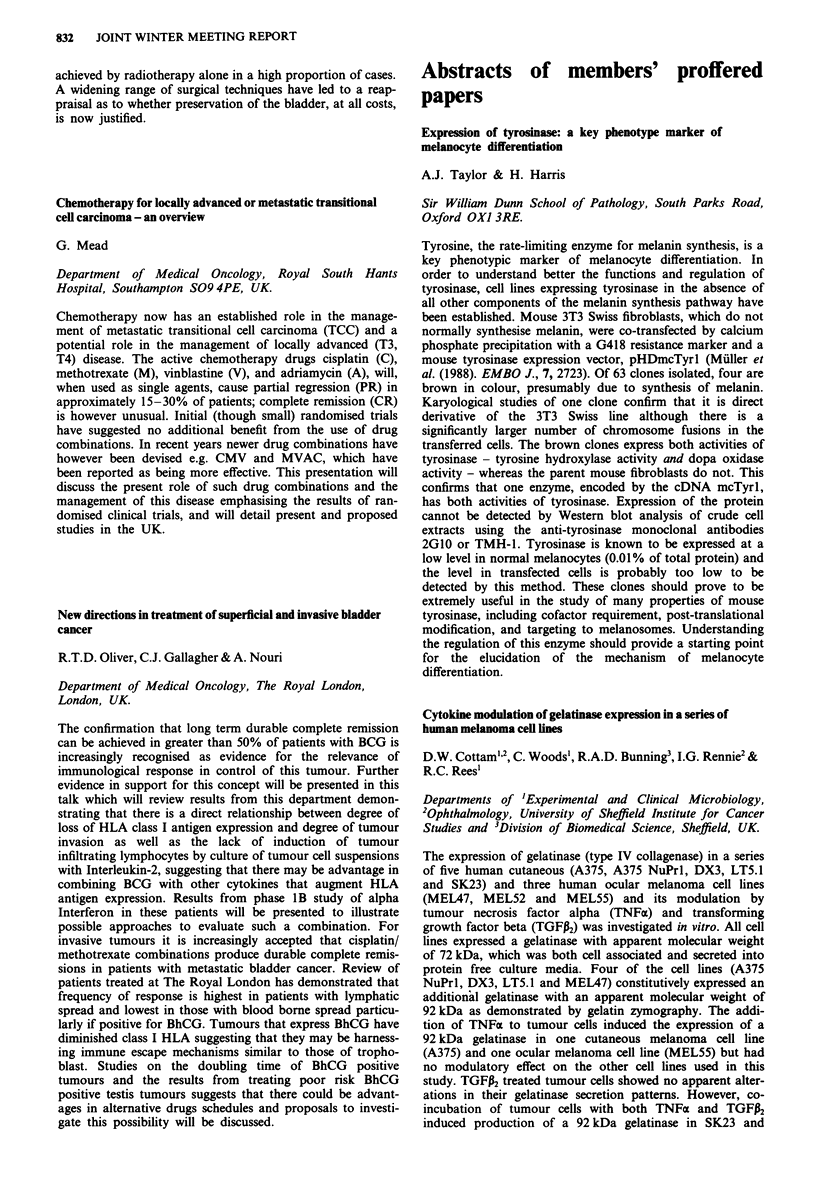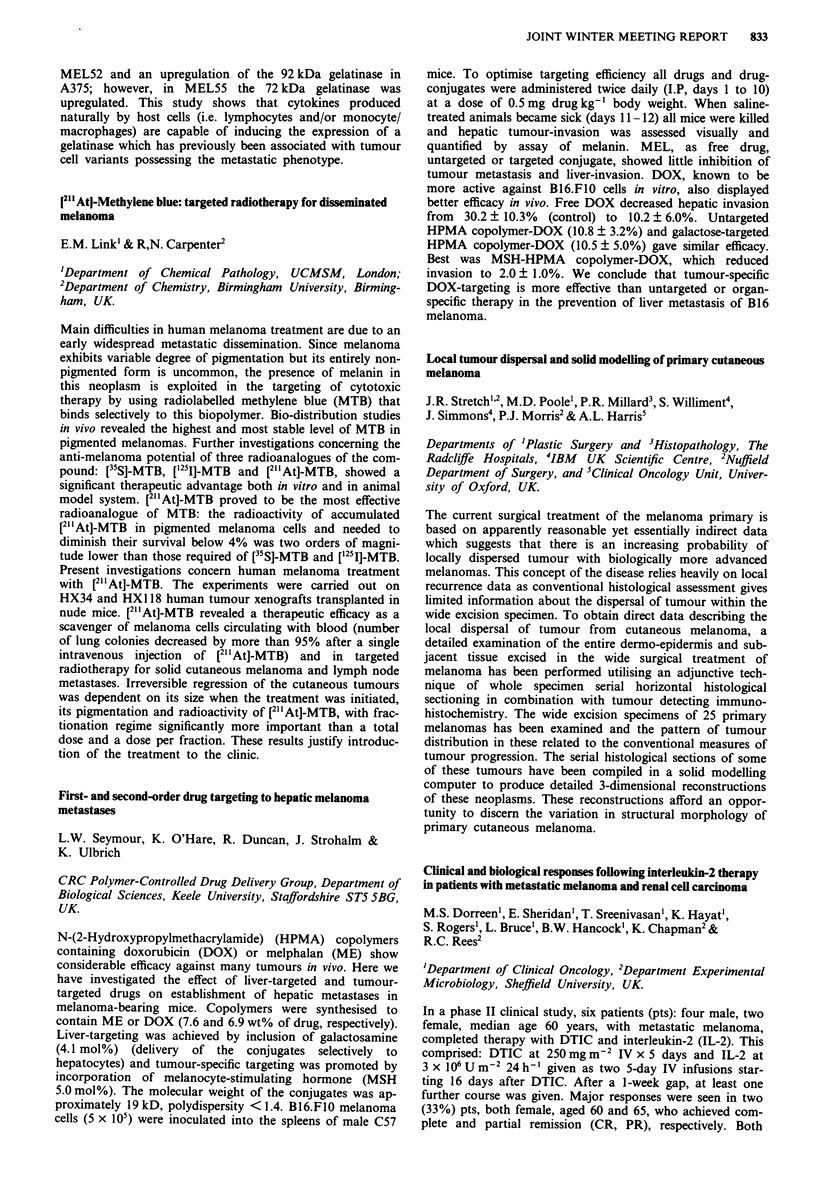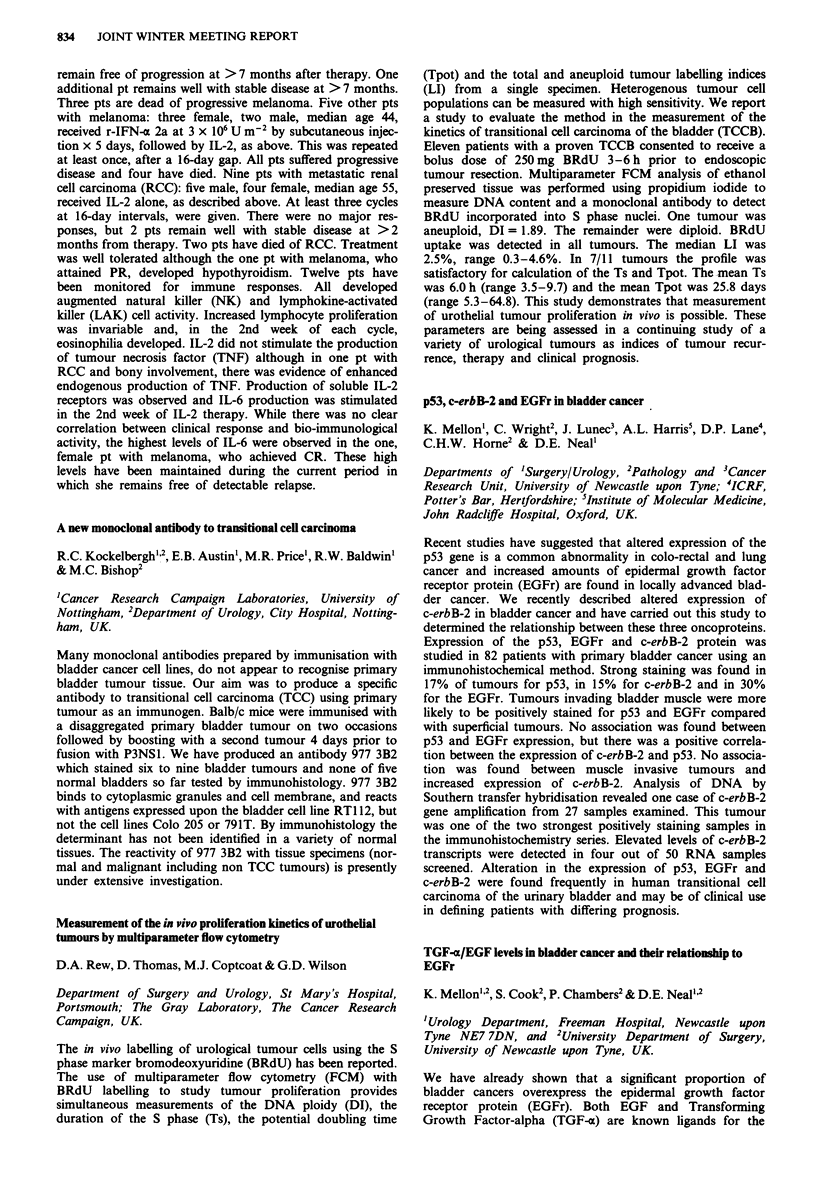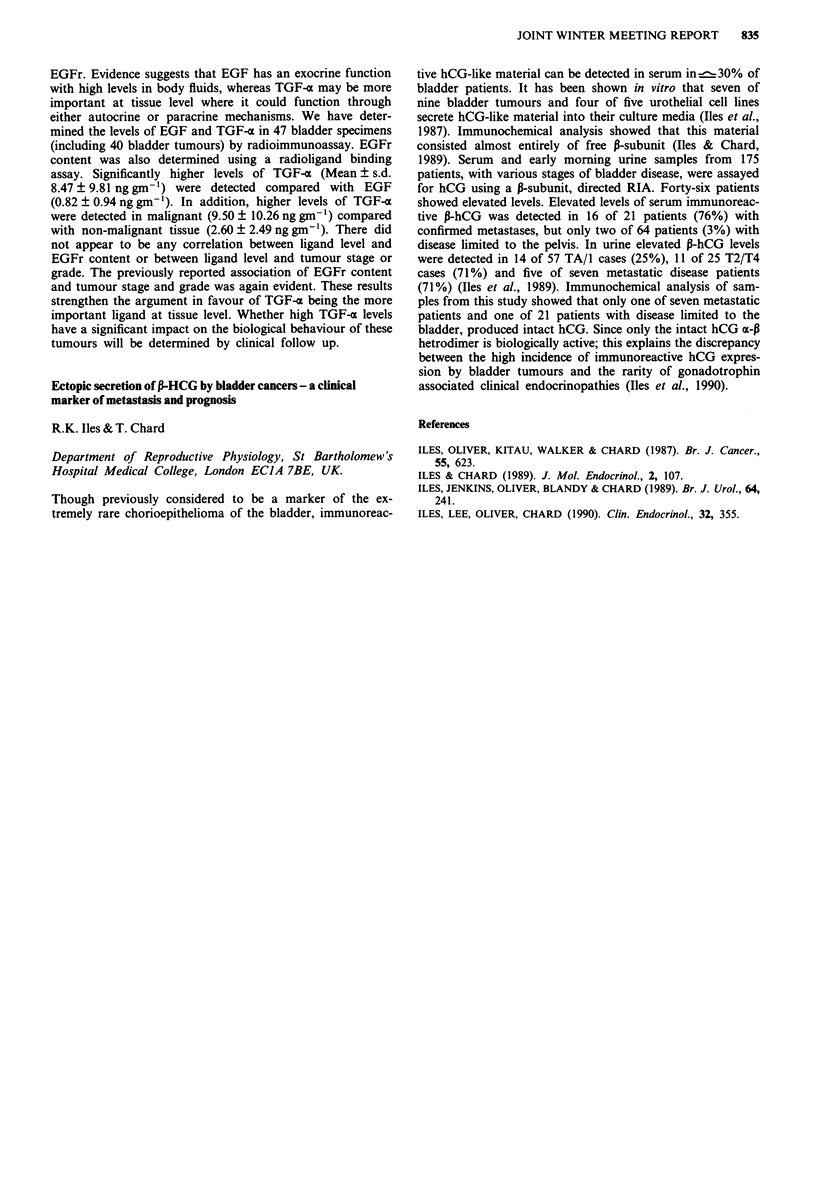# British Association for Cancer Research/Association of Cancer Physicians/Royal Society of Medicine (Oncology Section) Joint Winter Meeting

**Published:** 1991-05

**Authors:** 


Br. J. Cancer (1991), 63, 828-835 © Macmillan Press Ltd., 1991

MEETING REPORT

### British Association for Cancer Research/Association of Cancer Physicians/Royal Society of Medicine (Oncology Section) Joint Winter Meeting Including Symposia on 'Cutaneous Melanoma' (Incorporating the 11th Gordon Hamilton-Fairley Memorial Lecture) and 'Bladder Cancer' (Incorporating the 8th Alexander Haddow Memorial Lecture)

*Held at the Royal Society of Medicine, Wimpole Street, London W1, UK on 29-30 November 1990.*

### Abstracts of invited papers

### Regulatory factors in normal and malignant melanocytes

R. Halaban[1], Y. Funasaka[1], J. Cowan[2] & D. Birnbaum[3]

[1]*Department of Dermatology, Yale University School of Medicine, New Haven, Connecticut, USA;* [2]*Department of Radiation Oncology, University of Chicago Medical Center, Chicago, Illinois, USA;* [3]*Institute Paoli-Calmettes, Marseille, France.*

Melanomas are highly variable with respect to aberrant gene expression and chromosomal lesions but share a common characteristic of an acquired independence from environmental growth factors that are needed for proliferation of normal melanocytes. This autonomy is achieved in part via the endogenous production of basic fibroblast growth factor (bFGF), which is not expressed in normal melanocytes. Other melanocyte mitogens such as acidic FGF (aFGF), K-FGF/hst, FGF-5 and FGF-6, are not expressed in melanomas, in spite of the presence of amplified K-FGF/int-2 linked genes in one out of ten melanomas. These other mitogens may however, have a role in promoting the growth of melanomas at site of metastasis since some of them are known to be produced in normal tissues. The bFGF receptor is a tyrosine protein kinase that is constitutively active in melanomas. Blocking of the tyrosine-kinase activity by antagonists, antibodies or low molecular weight inhibitors, retards melanoma growth in culture, suggesting new directions for drug design for the clinical management of metastatic melanomas.

### Mesenchymal interactions with human melanoma cells affecting tumour cell growth are a function of tumour progression

I. Cornil & R.S. Kerbel

*Samuel Lunenfeld Research Institute, Mt. Sinai Hospital, 600 University Avenue, Toronto, Ontario M5G 1X5, Canada.*

It is known that the ability of tumour cells to grow locally and metastasise can be affected by the presence of adjacent normal tissues and cells, such as fibroblasts. However the comparative influence of such cell interactions on tumour behaviour has not been thoroughly investigated from the perspective of diffeent stages of tumour progression. Specifically, we wished to determine whether the nature and effects of such cell interactions would vary between metastatically-competent *vs* incompetent tumour cell populations. To address this question we assessed the influence of normal dermal fibroblasts on the growth of human melanoma cells obtained from different stages of tumour progression. We found that the *in vitro* growth of most (four out of five) melanoma cell lines derived from early stage metastatically-incompetent primary lesions is repressed by coculture with normal dermal fibroblasts, suggesting that negative homeostatic growth controls are still operative on melanoma cells from early stages of disease. On the other hand, nine out of 11 melanoma cell lines derived from advanced metastatically competent primary lesions, or from distant metastases, were found to be growth *stimulated* in the presence of dermal fibroblasts. This discriminatory fibroblastic influence is mediated by soluble inhibitory and stimulatory growth factor(s) whose nature could not be identified by using neutralising antibodies directed against known growth factors. Taken together, these results indicate that fibroblast-derived signals can have antithetical effects on metastatic *vs* metastatically-incompetent tumour subpopulations. This resultant conversion in responsiveness may confer upon metastatic cells a growth advantage allowing them to escape local growth controls both locally and at distant ectopic tissue sites.

### Changes in antigen expression during melanoma progression

M. Herlyn & I. Juhasz

*The Wistar Institute of Anatomy and Biology, Philadelphia, Pennsylvania 19104, USA.*

Expression of antigenic markers on different cell types of the melanocytic lineage changes with tumour progression. Few antigens have been identified with monoclonal antibodies that define only normal and non-malignant melanocytic cells. These include the adenosine deaminase binding protein and a 140,000 dalton glycoprotein. Higher expression of gangliosides $GD_3$ and acetylated $GD_3$, cell-cell interacting chondroitin sulfate proteoglycan gp34, and HLA-DR were found on mature nevus cells *vs* normal melanocytes. Differences between mature and dysplastic nevi were not significant nor were those between advanced (vertical growth phase [VGP]) primary and metastatic melanomas. Differences were most notable between non-invasive (radial growth phase [RGP])

The following companies and associations have been major contributors to this meeting: Amgen Roche, Cancer Research Campaign, Ciba-Geigy Pharmaceuticals, Imperial Cancer Research Fund. We are also grateful for contributions from: Boehringer Ingelheim, Dako Ltd, Glaxo Group Research Ltd, Nikon UK Ltd, Smithkline Beecham Pharmaceuticals.

and invasive (VGP) primary melanomas. When the results on dysplastic nevi and RGP primary melanomas were combined and compared with those of VGP primary melanomas and metastases, several antigens were expressed in significantly higher numbers on more malignant cells. These include the vitronectin receptor, the receptors for EGF and transferrin, the extracellular matrix protein tenascin, ganglioside $GD_2$ and the cell-cell adhesion molecules ICAM-1 and MUC18. These results indicate that extensive structural changes occur in cells with tumour progression.

### *De novo* expression of cell adhesion molecules correlates with the development of metastatic potential in cutaneous melanoma

J.P. Johnson, J.M. Lehmann, B.G. Stade & G. Riethmüller

*Institute for Immunology, University of Munich, Goethestrasse 31, 8000 Munich 2, Germany.*

Metastatic potential in cutaneous melanoma first appears with the onset of the vertical growth phase and the probability of developing metastatic disease increases with increasing vertical thickness of the tumour. Searching for changes in gene expression which correlate with increasing tumour thickness, may lead to the identification of molecules which are involved in the metastatic process. We isolated two monoclonal antibodies which reacted with more than 70% of thick primary melanomas, but which showed no reactivity with melanocytic nevi or with thin radial growth phase primary tumours. The cDNAs encoding these antigens were obtained by antibody screening of expression libraries. Sequencing of the cDNA clones revealed that one antigen was the intercellular adhesion molecule ICAM-1 which mediates adhesion with leukocytes. The second antigen, MUC18, is a novel member of the immunoglobulin superfamily and has structural characteristics of a cell adhesion molecule. Alterations in intercellular adhesion are thought to play a major role in the metastatic process and the role of these two molecules in the interaction of melanoma cells with other cells and tissues is being examined.

### 1990 Gordon Hamilton-Fairley Memorial Lecture

### The comparative biology of tumour progression

W.H. Clark Jr

*Pigmented Lesion Study Group, 217 Clinical Research Buildings, 422 Curle Boulevard, University of Pennsylvania, Philadelphia, Pennsylvania 19104, USA*:

Induction of a neoplastic system produces focal, proliferative lesions that are benign at the outset. Tumour progression is a sequence of changes occurring within the proliferative lesions of neoplasia resulting in a series of qualitatively different lesions; lesions that may progress from benign to malignant to increasing malignancy. Tumour progression is the biologic phenomenon linking the seemingly heterogeneous lesions of neoplasia into a single disease entity. Cancer is derived through tumour progression not by transformation of a putatively normal cell. Photographs of pigmented lesions of all patients seen over 14 years, histologic study of lesions from the same cohort of patients, and the *in vitro* study of cells derived from representative lesions of these patients have permitted us to define the lesions of tumour progression. Class I lesions are *precursor lesions* showing temporarily restricted growth confined to the tissue compartment of origin. Early Class I lesions are benign tumours that tend to differentiate and disappear. Later Class I lesions show aberrant differentiation and still later ones show dysplasia and may occasionally progress to Class II. Class II lesions do not metastasise and are intermediate between the precursor lesions of Class I and overt, invasive primary cancer, Class III. Neoplastic cell growth in Class II is temporarily unrestricted, but still confined to the original tissue compartment; invasion is not manifest. Class III lesions are invasive primary cancers. Early on such cancers lack capacity for metastasis, but through progression acquire metastatic competence. In melanoma, metastases are predicted by the nature of at least six attributes: mitotic rate $mm^{-2}$; quantity of tumour infiltrating lymphocytes; thickness; anatomic site of the primary; sex of the patient, and the presence of histologic regression. Class IV lesions are metastases. The study of cutaneous squamous cell carcinoma, colonic carcinoma and hepatocellular carcinoma has shown diverse neoplastic systems to be similar even when inductive mechanisms are apparently quite different.

### Aetiology and prevention of melanoma

R.M. MacKie

*Dermatology Department, University of Glasgow, Glasgow G12 8QQ, UK.*

Detailed epidemiological and case control studies from Australia, Western Canada, Denmark and Scotland strongly implicate exposure to ultraviolet radiation as a major aetiological factor in cutaneous malignant melanoma. For superficial spreading and nodular melanomas short episodes of intense UV exposure such as those encountered on continental holidays appear of particular importance, while for lentigo maligna melanoma total lifetime sun exposure appears more important. A large number of case control studies in the past 5 years have identified total number of melanocytic naevi as the strongest risk factor for melanoma, and there are at present several studies investigating the aetiology of naevi. In Scotland the four strongest risk factors in a large case control study are total number of naevi, presence of freckles, number of naevi of diameter 5 mm or greater and episodes of severe sunburn. Both primary and secondary prevention of melanoma are theoretically feasible. Secondary prevention involves public education to encourage referral and treatment of melanomas when they are in an earlier growth phase and thinner tumours. A recent Scottish study has recorded a statistically significant drop in melanoma Breslow thickness over a period of public education. The main thrust of primary prevention would appear to be encouraging a more cautious attitude to sun exposure and prevention of UV-induced damage. In Australia active public education has resulted in striking behavioural change. It will take some years to evaluate whether or not this is associated with a fall in melanoma incidence and, more important, mortality.

### Chemotherapy for melanoma

M.E. Gore

*Melanoma Unit, Royal Marsden Hospital, Fulham Road, London, UK.*

The results of chemotherapy for advanced melanoma are disappointing. The most active single agents are: dacarbazine (DTIC) which has a response rate of 24%, and vindesine, cisplatin and nitrosoureas which all have response rates of about 15%. Response durations are short, 4–6 months and complete remissions to single agent therapy rare. Randomised studies of DTIC – containing combinations or combinations without DTIC do not suggest any advantage over single agent DTIC. Response rates of almost 50% have been reported in some more recent phase II studies using DTIC – or cisplatin – containing regimens, but randomised trials are needed to properly evaluate these new schedules. Recently, there has been considerable interest in the observation that when tamoxifen is added to a combination of cisplatin, DTIC and BCNU the response rate is doubled. One of the major difficulties in interpreting the results of chemotherapy in melanoma is that different sites of disease show different rates of response: lymph node, skin or soft tissue metastases are the most responsive (34%) followed by the lungs (16%) and finally the brain and liver (8%). The same regimen may therefore have a very different response rate depending on the proportion of patients that have a visceral element to their metastatic disease. There is no evidence that chemotherapy is of any benefit when given in the adjuvant setting to patients at high risk from relapse from melanoma. Future studies are likely to involve the integration of chemotherapy with biological response modifiers such as interferon and interleukin-2 and these approaches are already showing promise.

### Recombination interleukin 2 (IL-2) in advanced malignant melanoma

N. Thatcher

*Department of Medical Oncology, Christie Hospital, Manchester M20 9BX, UK.*

IL-2 a glycoprotein secreted by activated T lymphocytes is a growth factor for antigen stimulated T cells. IL-2 also stimulates the production of other cytokines which may themselves have antitumour activity. The tumourcidal activity of natural killer (NK) cells and cytotoxic T lymphocytes is also enhanced by IL-2 and these lymphokine activated killer (LAK) cells are capable of lysing fresh human tumour cells. Rosenberg and colleagues have recently updated the original 1985 study of IL-2 + LAK cell (generated *ex vivo*) therapy. An objective response rate of 21% in 48 patients with advanced melanoma was reported with 5–10% having durable remissions for up to 4 years (*Ann. Surg.*, **210**, 474, 1989). Subsequent confirmation of antimelanoma activity came from the IL-2/LAK extramural working group (Dutcher *et al.*, *J. Clin. Oncol.*, **7**, 477, 1989). These early studies used the maximum tolerated doses of IL-2 and were associated with severe toxicity in some patients requiring ICU facilities. The use of continuous infusion or intermittent i.v. bolus IL-2 therapy has allowed similar total doses to be delivered as by the Rosenberg group but with much less toxicity and similar antimelanoma activity (West *et al.*, *New Engl. J. Med.*, **316**, 898, 1987; Thatcher *et al.*, *Br. J. Cancer.*, **60**, 770, 1989). IL-2 alone may indeed be as effective as the IL-2 + LAK cell combination and avoids the considerable logistic difficulties of LAK cell generation. IL-2 has also been used in combination with chemotherapy e.g. cyclophosphamide at low dose as an immunomodulating agent or with DTIC as a cytotoxic agent. Other approaches have included the expansion of tumour infiltrating lymphocytes (TIL) and of more general interest IL-2 with Interferons. The latter combination may provide greater antimelanoma activity than either agent alone. Recently the feasibility of out patient therapy for this combination of cytokines has been established and little toxicity has been observed. IL-2 is therefore an important addition to the treatment of advanced melanoma and has established immunotherapy as the fourth modality of cancer therapy.

### Active specific immunotherapy with allogeneic melanoma lysates

M.S. Mitchell

*Departments of Medicine and Microbiology, University of Southern California Cancer Center, Los Angeles, California 90033, USA.*

In order to validate the efficacy of our materials prior to treating minimal residual disease, we have treated patients with measurable disseminated melanoma, with active specific immunotherapy. Mechanical lysates of two cell lines of melanoma were combined with the adjuvant DETOX and injected s.c. on weeks 1, 2, 3, 4 and 6 into nearly 100 patients in six separate trials. A major response rate of 20% has been achieved, with 5% complete remissions. Another 10% of the patients have had minor responses: (25–50% shrinkage for >4 week or >50% shrinkage lasting <4 weeks). Complete or partial remissions have been seen in subcutaneous sites, lymph nodes, lungs, and small intestine, with 33% shrinkage of a large liver nodule in one patient and disappearance of a 1.5 cm liver nodule (cyst?) in another. Thus far, seven patients have had continuing responses lasting 1 to >4 years, on 'maintenance' immunotherapy. One patient with a *primary* ocular melanoma has also been treated. The lesion shrank from 4.2 to 2.3 mm within 6 weeks and has remained that size for >6 months. No toxicity was associated with this treatment, except local granulomas in patients receiving 15 or more injections. The frequency of cytolytic T cell precursors has been increased in responders, whereas patients without an increase invariably have failed to respond. Both CD4 + and CD8 + cytolytic T cell clones have been grown from treated patients. Most CD4 + CTL were not Class II MHC restricted, reacting broadly against melanoma cell lines but not other types of tumour. Several of the clones in fact reacted against melanomas lacking Class II MHC antigens. Some CD8 + CTL were self-MHC restricted, as in mice, but more usually recognised melanoma antigens presented by the HLA-A2 molecule on several cell lines. The HLA phenotypes of responders and non-responders were compared. Significant correlations have been found between clinical response and the presence of HLA-A2 together with either B44 or DR4. Allogeneic lysates and DETOX may have stimulated additional T-helper cells and augmented the response to autologous melanoma-associated antigens, leading to responses in genetically favourable individuals. A large-scale clinical trial randomising patients to treatment with melanoma theraccine or observation will now be performed in the setting of minimal residual disease.

### Prognostic factors in bladder cancer

C. Fisher

*Department of Histopathology, Royal Marsden Hospital, Fulham Road, London SW3 6JJ, UK.*

Transitional cell carcinomas account for about 95% of bladder carcinomas in the UK. They are separable into subtypes with different behaviour and management: carcinoma *in situ* (CIS), superficial disease and muscle-invasive carcinoma. A principal prognostic factor is T stage, for which the preferred staging system remains the the UICC 3rd Edition (1978) rather than the 4th Edition (1987). The so-called muscularis mucosae may form a prognostic subdividing line within T1 tumours. For superficial tumours, factors reported as predisposing to recurrence or progression include number and size of tumours, grade, coexistent CIS, cell surface blood group antigen status, aneuploidy, EGF receptor expression, and findings at 3 month follow up cystoscopy. Behaviour of muscle-invasive tumours may be influenced by tumour size, grade, pattern of invasion, lymphatic invasion and lymph nodal status. The presence of squamous metaplasia may affect response to radiotherapy. For other types of bladder carcinoma the prognosis is related principally to stage at presentation (squamous cell or adenocarcinoma), or to histological subtype (small cell).

**Molecular biology of bladder cancer**

M.A. Knowles, J.P. Cairns, L.M. Coombs & A.J. Proctor

*Marie Curie Research Institute, The Chart, Oxted, Surrey RH8 0TL, UK.*

A number of genetic changes are required for epithelial transformation. For several tumour types including colon, breast and lung, the identity of certain of the genes involved is now known, some of the molecular mechanisms involved in the generation of the transformed phenotype have been elucidated and clinical correlates with these molecular changes have been identified. Some changes are shared by several tumour types whilst others appear to be cell type specific. Recently, a number of frequent molecular alterations have been identified in transitional cell tumours. We have shown that the proto-oncogene HER2 is amplified and over-expressed in many tumours and that this shows a strong correlation with tumour grade. Amplification of HER2 was detected in 2% of grade 1, 16% of grade 2 and 46% of grade 3 tumours. The protein product of this gene can be detected by immunohistochemistry only at the luminal surface of mature superficial cells in the normal urothelium but shows a widespread distribution in some tumours. We have also identified a region of amplified DNA on chromosome 11q13 in 20% of tumours. This region contains the oncogenes INT2 and HST, members of the FGF family of genes and the BCL1 locus which is frequently involved in breakpoints in B cell tumours. The identity of the target gene within this amplicon is not known. Since expression of INT2 and HST are not detected in tumours with gene amplification, it is postulated that an unknown gene within this region has yet to be identified. These amplifications show no correlation with tumour grade, nor with HER2 amplification status. A third genetic change recently identified in this laboratory concerns the retinoblastoma susceptibility gene RB1. In 30% of bladder tumours we have detected loss or re-arrangement of one allele of the gene. These changes appear to involve predominantly tumours of high grade and stage. Our findings, together with those from other laboratories including the presence of RAS mutations, p53 mutations, over-expression of the epidermal growth factor receptor and the identification of sites of allelic loss on chromosomes 9q, 11p and 17p now provide the basis for a classification of transitional cell tumours according to the genetic lesions they contain. The divergent clinical phenotype of bladder tumours is well known. It may be postulated that the number, combination and/or timing of such molecular events determines the natural history of the disease and the phenotype of the tumours which develop. The identification of these lesions in bladder tumours should therefore present powerful diagnostic and prognostic tools and may provide the key to progress in therapy.

**Intravesical Bacillus calmette-guerin for carcinoma *in situ* of the urinary bladder**

D.M.A. Wallace

*The Queen Elizabeth Hospital, Birmingham, UK.*

BCG was first instilled into the bladder in the early 1970's as empirical immunotherapy for superficial bladder tumours. It has since been shown to be as effective as intravesical chemotherapy in the treatment of superficial tumours and in prophylaxsis against recurrences. Some studies have also shown complete response rates for carcinoma *in situ* of the bladder (CIS) of up to 90% which have been sustained. CIS of the bladder remains a disease that is hard to define and quantify. Few randomised trials of BCG in CIS of the bladder are available. The side effects of BCG can be serious and sometimes fatal. Several different strains are in use at present and a recent MRC Study suggests that there may be differences in side effects and in efficacy which will require a larger study to demonstrate if these are significant. The exact mode of action of BCG is not clear and the optimum treatment schedule for each strain is yet to be established. Efforts must continue to determine the appropriate end point of treatment in order to reduce the local and systemic toxicity of intravesical BCG.

**The role of radiotherapy in the treatment of bladder cancer**

J.T. Roberts

*Regional Radiotherapy Centre, Newcastle General Hospital, Newcastle upon Tyne, UK.*

Modern megavoltage radiotherapy can cure some patients with muscle-invasive bladder cancer, producing an overall 5-year survival of 17–39% in a number of series, with preservation of a functioning bladder. The identification of appropriate prognostic indicators such as patholgoical subtypes, radiographic appearance, clinical stage and radiation responsiveness may allow the selection of those most likely to benefit from radical radiotherapy. Radiotherapy alone produces immediate complete response rates of approximately 50% in the bladder. The observation, by several workers, of immediate complete response rates of 70% or more following combined therapy with radiation and cisplatin-based chemotherapy regimes has led to the instigation of a number of controlled trials of combination therapy. Recently published retrospective studies have suggested that radical external beam irradiation may have a role in the management of high grade superficial bladder cancer and the Medical Research Council are proposing a randomised study to assess this role.

### 1990 Alexander Haddow Memorial Lecture

**Bladder cancer: the integration of surgery with other modalities**

G.D. Chisholm

*University Department of Surgery/Urology, Western General Hospital, Edinburgh EH4 2XU, UK.*

Although it might seem the surgical extirpation of bladder cancer should be followed by a very good survival rate, the facts are that this is not necessarily so. Superficial bladder cancers, 'completely' removed by surgical methods, have about a 75% 5-year survival rate. Invasive bladder cancers, 'completely' removed by radical excision have about a 35% 5-year survival rate. Thus it is evident that surgery, at best, can only be part of an overall management strategy. Because superficial bladder cancer is a multifocal disease, most cases require additional treatment in the hope of preventing recurrence and arresting progression. The management of invasive bladder cancer remains a frustration because methods for detecting distant spread are crude. Where selection methods are optimum then the results of monotherapy may be improved, to a degree, but overall the management of invasive bladder cancer should now incorporate one of the combinations – surgery/radiotherapy/chemotherapy. Protagonists of radiotherapy have a long history in UK urology based mainly on the hope that preservation of the bladder could be achieved by radiotherapy alone in a high proportion of cases. A widening range of surgical techniques have led to a reappraisal as to whether preservation of the bladder, at all costs, is now justified.

**Chemotherapy for locally advanced or metastatic transitional cell carcinoma – an overview**

G. Mead

*Department of Medical Oncology, Royal South Hants Hospital, Southampton SO9 4PE, UK.*

Chemotherapy now has an established role in the management of metastatic transitional cell carcinoma (TCC) and a potential role in the management of locally advanced (T3, T4) disease. The active chemotherapy drugs cisplatin (C), methotrexate (M), vinblastine (V), and adriamycin (A), will, when used as single agents, cause partial regression (PR) in approximately 15–30% of patients; complete remission (CR) is however unusual. Initial (though small) randomised trials have suggested no additional benefit from the use of drug combinations. In recent years newer drug combinations have however been devised e.g. CMV and MVAC, which have been reported as being more effective. This presentation will discuss the present role of such drug combinations and the management of this disease emphasising the results of randomised clinical trials, and will detail present and proposed studies in the UK.

**New directions in treatment of superficial and invasive bladder cancer**

R.T.D. Oliver, C.J. Gallagher & A. Nouri

*Department of Medical Oncology, The Royal London, London, UK.*

The confirmation that long term durable complete remission can be achieved in greater than 50% of patients with BCG is increasingly recognised as evidence for the relevance of immunological response in control of this tumour. Further evidence in support for this concept will be presented in this talk which will review results from this department demonstrating that there is a direct relationship between degree of loss of HLA class I antigen expression and degree of tumour invasion as well as the lack of induction of tumour infiltrating lymphocytes by culture of tumour cell suspensions with Interleukin-2, suggesting that there may be advantage in combining BCG with other cytokines that augment HLA antigen expression. Results from phase 1B study of alpha Interferon in these patients will be presented to illustrate possible approaches to evaluate such a combination. For invasive tumours it is increasingly accepted that cisplatin/methotrexate combinations produce durable complete remissions in patients with metastatic bladder cancer. Review of patients treated at The Royal London has demonstrated that frequency of response is highest in patients with lymphatic spread and lowest in those with blood borne spread particularly if positive for BhCG. Tumours that express BhCG have diminished class I HLA suggesting that they may be harnessing immune escape mechanisms similar to those of trophoblast. Studies on the doubling time of BhCG positive tumours and the results from treating poor risk BhCG positive testis tumours suggests that there could be advantages in alternative drugs schedules and proposals to investigate this possibility will be discussed.

### Abstracts of members' proffered papers

**Expression of tyrosinase: a key phenotype marker of melanocyte differentiation**

A.J. Taylor & H. Harris

*Sir William Dunn School of Pathology, South Parks Road, Oxford OX1 3RE.*

Tyrosine, the rate-limiting enzyme for melanin synthesis, is a key phenotypic marker of melanocyte differentiation. In order to understand better the functions and regulation of tyrosinase, cell lines expressing tyrosinase in the absence of all other components of the melanin synthesis pathway have been established. Mouse 3T3 Swiss fibroblasts, which do not normally synthesise melanin, were co-transfected by calcium phosphate precipitation with a G418 resistance marker and a mouse tyrosinase expression vector, pHDmcTyr1 (Müller *et al.* (1988). *EMBO J.*, **7**, 2723). Of 63 clones isolated, four are brown in colour, presumably due to synthesis of melanin. Karyological studies of one clone confirm that it is direct derivative of the 3T3 Swiss line although there is a significantly larger number of chromosome fusions in the transferred cells. The brown clones express both activities of tyrosinase – tyrosine hydroxylase activity *and* dopa oxidase activity – whereas the parent mouse fibroblasts do not. This confirms that one enzyme, encoded by the cDNA mcTyr1, has both activities of tyrosinase. Expression of the protein cannot be detected by Western blot analysis of crude cell extracts using the anti-tyrosinase monoclonal antibodies 2G10 or TMH-1. Tyrosinase is known to be expressed at a low level in normal melanocytes (0.01% of total protein) and the level in transfected cells is probably too low to be detected by this method. These clones should prove to be extremely useful in the study of many properties of mouse tyrosinase, including cofactor requirement, post-translational modification, and targeting to melanosomes. Understanding the regulation of this enzyme should provide a starting point for the elucidation of the mechanism of melanocyte differentiation.

**Cytokine modulation of gelatinase expression in a series of human melanoma cell lines**

D.W. Cottam[1,2], C. Woods[1], R.A.D. Bunning[3], I.G. Rennie[2] & R.C. Rees[1]

*Departments of [1]Experimental and Clinical Microbiology, [2]Ophthalmology, University of Sheffield Institute for Cancer Studies and [3]Division of Biomedical Science, Sheffield, UK.*

The expression of gelatinase (type IV collagenase) in a series of five human cutaneous (A375, A375 NuPr1, DX3, LT5.1 and SK23) and three human ocular melanoma cell lines (MEL47, MEL52 and MEL55) and its modulation by tumour necrosis factor alpha (TNF$\alpha$) and transforming growth factor beta (TGF$\beta_2$) was investigated *in vitro*. All cell lines expressed a gelatinase with apparent molecular weight of 72 kDa, which was both cell associated and secreted into protein free culture media. Four of the cell lines (A375 NuPr1, DX3, LT5.1 and MEL47) constitutively expressed an additional gelatinase with an apparent molecular weight of 92 kDa as demonstrated by gelatin zymography. The addition of TNF$\alpha$ to tumour cells induced the expression of a 92 kDa gelatinase in one cutaneous melanoma cell line (A375) and one ocular melanoma cell line (MEL55) but had no modulatory effect on the other cell lines used in this study. TGF$\beta_2$ treated tumour cells showed no apparent alterations in their gelatinase secretion patterns. However, co-incubation of tumour cells with both TNF$\alpha$ and TGF$\beta_2$ induced production of a 92 kDa gelatinase in SK23 and

MEL52 and an upregulation of the 92 kDa gelatinase in A375; however, in MEL55 the 72 kDa gelatinase was upregulated. This study shows that cytokines produced naturally by host cells (i.e. lymphocytes and/or monocyte/macrophages) are capable of inducing the expression of a gelatinase which has previously been associated with tumour cell variants possessing the metastatic phenotype.

### [$^{211}$At]-Methylene blue: targeted radiotherapy for disseminated melanoma

E.M. Link[1] & R,N. Carpenter[2]

*[1]Department of Chemical Pathology, UCMSM, London; [2]Department of Chemistry, Birmingham University, Birmingham, UK.*

Main difficulties in human melanoma treatment are due to an early widespread metastatic dissemination. Since melanoma exhibits variable degree of pigmentation but its entirely non-pigmented form is uncommon, the presence of melanin in this neoplasm is exploited in the targeting of cytotoxic therapy by using radiolabelled methylene blue (MTB) that binds selectively to this biopolymer. Bio-distribution studies *in vivo* revealed the highest and most stable level of MTB in pigmented melanomas. Further investigations concerning the anti-melanoma potential of three radioanalogues of the compound: [$^{35}$S]-MTB, [$^{125}$I]-MTB and [$^{211}$At]-MTB, showed a significant therapeutic advantage both *in vitro* and in animal model system. [$^{211}$At]-MTB proved to be the most effective radioanalogue of MTB: the radioactivity of accumulated [$^{211}$At]-MTB in pigmented melanoma cells and needed to diminish their survival below 4% was two orders of magnitude lower than those required of [$^{35}$S]-MTB and [$^{125}$I]-MTB. Present investigations concern human melanoma treatment with [$^{211}$At]-MTB. The experiments were carried out on HX34 and HX118 human tumour xenografts transplanted in nude mice. [$^{211}$At]-MTB revealed a therapeutic efficacy as a scavenger of melanoma cells circulating with blood (number of lung colonies decreased by more than 95% after a single intravenous injection of [$^{211}$At]-MTB) and in targeted radiotherapy for solid cutaneous melanoma and lymph node metastases. Irreversible regression of the cutaneous tumours was dependent on its size when the treatment was initiated, its pigmentation and radioactivity of [$^{211}$At]-MTB, with fractionation regime significantly more important than a total dose and a dose per fraction. These results justify introduction of the treatment to the clinic.

### First- and second-order drug targeting to hepatic melanoma metastases

L.W. Seymour, K. O'Hare, R. Duncan, J. Strohalm & K. Ulbrich

*CRC Polymer-Controlled Drug Delivery Group, Department of Biological Sciences, Keele University, Staffordshire ST5 5BG, UK.*

N-(2-Hydroxypropylmethacrylamide) (HPMA) copolymers containing doxorubicin (DOX) or melphalan (ME) show considerable efficacy against many tumours *in vivo*. Here we have investigated the effect of liver-targeted and tumour-targeted drugs on establishment of hepatic metastases in melanoma-bearing mice. Copolymers were synthesised to contain ME or DOX (7.6 and 6.9 wt% of drug, respectively). Liver-targeting was achieved by inclusion of galactosamine (4.1 mol%) (delivery of the conjugates selectively to hepatocytes) and tumour-specific targeting was promoted by incorporation of melanocyte-stimulating hormone (MSH 5.0 mol%). The molecular weight of the conjugates was approximately 19 kD, polydispersity $<1.4$. B16.F10 melanoma cells ($5 \times 10^5$) were inoculated into the spleens of male C57 mice. To optimise targeting efficiency all drugs and drug-conjugates were administered twice daily (I.P, days 1 to 10) at a dose of 0.5 mg drug $kg^{-1}$ body weight. When saline-treated animals became sick (days 11–12) all mice were killed and hepatic tumour-invasion was assessed visually and quantified by assay of melanin. MEL, as free drug, untargeted or targeted conjugate, showed little inhibition of tumour metastasis and liver-invasion. DOX, known to be more active against B16.F10 cells *in vitro*, also displayed better efficacy *in vivo*. Free DOX decreased hepatic invasion from $30.2 \pm 10.3\%$ (control) to $10.2 \pm 6.0\%$. Untargeted HPMA copolymer-DOX ($10.8 \pm 3.2\%$) and galactose-targeted HPMA copolymer-DOX ($10.5 \pm 5.0\%$) gave similar efficacy. Best was MSH-HPMA copolymer-DOX, which reduced invasion to $2.0 \pm 1.0\%$. We conclude that tumour-specific DOX-targeting is more effective than untargeted or organ-specific therapy in the prevention of liver metastasis of B16 melanoma.

### Local tumour dispersal and solid modelling of primary cutaneous melanoma

J.R. Stretch[1,2], M.D. Poole[1], P.R. Millard[3], S. Williment[4], J. Simmons[4], P.J. Morris[2] & A.L. Harris[5]

*Departments of [1]Plastic Surgery and [3]Histopathology, The Radcliffe Hospitals, [4]IBM UK Scientific Centre, [2]Nuffield Department of Surgery, and [5]Clinical Oncology Unit, University of Oxford, UK.*

The current surgical treatment of the melanoma primary is based on apparently reasonable yet essentially indirect data which suggests that there is an increasing probability of locally dispersed tumour with biologically more advanced melanomas. This concept of the disease relies heavily on local recurrence data as conventional histological assessment gives limited information about the dispersal of tumour within the wide excision specimen. To obtain direct data describing the local dispersal of tumour from cutaneous melanoma, a detailed examination of the entire dermo-epidermis and subjacent tissue excised in the wide surgical treatment of melanoma has been performed utilising an adjunctive technique of whole specimen serial horizontal histological sectioning in combination with tumour detecting immunohistochemistry. The wide excision specimens of 25 primary melanomas has been examined and the pattern of tumour distribution in these related to the conventional measures of tumour progression. The serial histological sections of some of these tumours have been compiled in a solid modelling computer to produce detailed 3-dimensional reconstructions of these neoplasms. These reconstructions afford an opportunity to discern the variation in structural morphology of primary cutaneous melanoma.

### Clinical and biological responses following interleukin-2 therapy in patients with metastatic melanoma and renal cell carcinoma

M.S. Dorreen[1], E. Sheridan[1], T. Sreenivasan[1], K. Hayat[1], S. Rogers[1], L. Bruce[1], B.W. Hancock[1], K. Chapman[2] & R.C. Rees[2]

*[1]Department of Clinical Oncology, [2]Department Experimental Microbiology, Sheffield University, UK.*

In a phase II clinical study, six patients (pts): four male, two female, median age 60 years, with metastatic melanoma, completed therapy with DTIC and interleukin-2 (IL-2). This comprised: DTIC at 250 mg $m^{-2}$ IV $\times$ 5 days and IL-2 at $3 \times 10^6$ U $m^{-2}$ 24 $h^{-1}$ given as two 5-day IV infusions starting 16 days after DTIC. After a 1-week gap, at least one further course was given. Major responses were seen in two (33%) pts, both female, aged 60 and 65, who achieved complete and partial remission (CR, PR), respectively. Both remain free of progression at $>7$ months after therapy. One additional pt remains well with stable disease at $>7$ months. Three pts are dead of progressive melanoma. Five other pts with melanoma: three female, two male, median age 44, received r-IFN-α 2a at $3\times10^6$ U $m^{-2}$ by subcutaneous injection × 5 days, followed by IL-2, as above. This was repeated at least once, after a 16-day gap. All pts suffered progressive disease and four have died. Nine pts with metastatic renal cell carcinoma (RCC): five male, four female, median age 55, received IL-2 alone, as described above. At least three cycles at 16-day intervals, were given. There were no major responses, but 2 pts remain well with stable disease at $>2$ months from therapy. Two pts have died of RCC. Treatment was well tolerated although the one pt with melanoma, who attained PR, developed hypothyroidism. Twelve pts have been monitored for immune responses. All developed augmented natural killer (NK) and lymphokine-activated killer (LAK) cell activity. Increased lymphocyte proliferation was invariable and, in the 2nd week of each cycle, eosinophilia developed. IL-2 did not stimulate the production of tumour necrosis factor (TNF) although in one pt with RCC and bony involvement, there was evidence of enhanced endogenous production of TNF. Production of soluble IL-2 receptors was observed and IL-6 production was stimulated in the 2nd week of IL-2 therapy. While there was no clear correlation between clinical response and bio-immunological activity, the highest levels of IL-6 were observed in the one, female pt with melanoma, who achieved CR. These high levels have been maintained during the current period in which she remains free of detectable relapse.

### A new monoclonal antibody to transitional cell carcinoma

R.C. Kockelbergh[1,2], E.B. Austin[1], M.R. Price[1], R.W. Baldwin[1] & M.C. Bishop[2]

*[1]Cancer Research Campaign Laboratories, University of Nottingham, [2]Department of Urology, City Hospital, Nottingham, UK.*

Many monoclonal antibodies prepared by immunisation with bladder cancer cell lines, do not appear to recognise primary bladder tumour tissue. Our aim was to produce a specific antibody to transitional cell carcinoma (TCC) using primary tumour as an immunogen. Balb/c mice were immunised with a disaggregated primary bladder tumour on two occasions followed by boosting with a second tumour 4 days prior to fusion with P3NS1. We have produced an antibody 977 3B2 which stained six to nine bladder tumours and none of five normal bladders so far tested by immunohistology. 977 3B2 binds to cytoplasmic granules and cell membrane, and reacts with antigens expressed upon the bladder cell line RT112, but not the cell lines Colo 205 or 791T. By immunohistology the determinant has not been identified in a variety of normal tissues. The reactivity of 977 3B2 with tissue specimens (normal and malignant including non TCC tumours) is presently under extensive investigation.

### Measurement of the *in vivo* proliferation kinetics of urothelial tumours by multiparameter flow cytometry

D.A. Rew, D. Thomas, M.J. Coptcoat & G.D. Wilson

*Department of Surgery and Urology, St Mary's Hospital, Portsmouth; The Gray Laboratory, The Cancer Research Campaign, UK.*

The *in vivo* labelling of urological tumour cells using the S phase marker bromodeoxyuridine (BRdU) has been reported. The use of multiparameter flow cytometry (FCM) with BRdU labelling to study tumour proliferation provides simultaneous measurements of the DNA ploidy (DI), the duration of the S phase (Ts), the potential doubling time (Tpot) and the total and aneuploid tumour labelling indices (LI) from a single specimen. Heterogenous tumour cell populations can be measured with high sensitivity. We report a study to evaluate the method in the measurement of the kinetics of transitional cell carcinoma of the bladder (TCCB). Eleven patients with a proven TCCB consented to receive a bolus dose of 250 mg BRdU 3–6 h prior to endoscopic tumour resection. Multiparameter FCM analysis of ethanol preserved tissue was performed using propidium iodide to measure DNA content and a monoclonal antibody to detect BRdU incorporated into S phase nuclei. One tumour was aneuploid, DI = 1.89. The remainder were diploid. BRdU uptake was detected in all tumours. The median LI was 2.5%, range 0.3–4.6%. In 7/11 tumours the profile was satisfactory for calculation of the Ts and Tpot. The mean Ts was 6.0 h (range 3.5–9.7) and the mean Tpot was 25.8 days (range 5.3–64.8). This study demonstrates that measurement of urothelial tumour proliferation *in vivo* is possible. These parameters are being assessed in a continuing study of a variety of urological tumours as indices of tumour recurrence, therapy and clinical prognosis.

### p53, c-*erb*B-2 and EGFr in bladder cancer

K. Mellon[1], C. Wright[2], J. Lunec[3], A.L. Harris[5], D.P. Lane[4], C.H.W. Horne[2] & D.E. Neal[1]

*Departments of [1]Surgery/Urology, [2]Pathology and [3]Cancer Research Unit, University of Newcastle upon Tyne; [4]ICRF, Potter's Bar, Hertfordshire; [5]Institute of Molecular Medicine, John Radcliffe Hospital, Oxford, UK.*

Recent studies have suggested that altered expression of the p53 gene is a common abnormality in colo-rectal and lung cancer and increased amounts of epidermal growth factor receptor protein (EGFr) are found in locally advanced bladder cancer. We recently described altered expression of c-*erb*B-2 in bladder cancer and have carried out this study to determined the relationship between these three oncoproteins. Expression of the p53, EGFr and c-*erb*B-2 protein was studied in 82 patients with primary bladder cancer using an immunohistochemical method. Strong staining was found in 17% of tumours for p53, in 15% for c-*erb*B-2 and in 30% for the EGFr. Tumours invading bladder muscle were more likely to be positively stained for p53 and EGFr compared with superficial tumours. No association was found between p53 and EGFr expression, but there was a positive correlation between the expression of c-*erb*B-2 and p53. No association was found between muscle invasive tumours and increased expression of c-*erb*B-2. Analysis of DNA by Southern transfer hybridisation revealed one case of c-*erb*B-2 gene amplification from 27 samples examined. This tumour was one of the two strongest positively staining samples in the immunohistochemistry series. Elevated levels of c-*erb*B-2 transcripts were detected in four out of 50 RNA samples screened. Alteration in the expression of p53, EGFr and c-*erb*B-2 were found frequently in human transitional cell carcinoma of the urinary bladder and may be of clinical use in defining patients with differing prognosis.

### TGF-α/EGF levels in bladder cancer and their relationship to EGFr

K. Mellon[1,2], S. Cook[2], P. Chambers[2] & D.E. Neal[1,2]

*[1]Urology Department, Freeman Hospital, Newcastle upon Tyne NE7 7DN, and [2]University Department of Surgery, University of Newcastle upon Tyne, UK.*

We have already shown that a significant proportion of bladder cancers overexpress the epidermal growth factor receptor protein (EGFr). Both EGF and Transforming Growth Factor-alpha (TGF-α) are known ligands for the

EGFr. Evidence suggests that EGF has an exocrine function with high levels in body fluids, whereas TGF-α may be more important at tissue level where it could function through either autocrine or paracrine mechanisms. We have determined the levels of EGF and TGF-α in 47 bladder specimens (including 40 bladder tumours) by radioimmunoassay. EGFr content was also determined using a radioligand binding assay. Significantly higher levels of TGF-α (Mean ± s.d. 8.47 ± 9.81 ng $gm^{-1}$) were detected compared with EGF (0.82 ± 0.94 ng $gm^{-1}$). In addition, higher levels of TGF-α were detected in malignant (9.50 ± 10.26 ng $gm^{-1}$) compared with non-malignant tissue (2.60 ± 2.49 ng $gm^{-1}$). There did not appear to be any correlation between ligand level and EGFr content or between ligand level and tumour stage or grade. The previously reported association of EGFr content and tumour stage and grade was again evident. These results strengthen the argument in favour of TGF-α being the more important ligand at tissue level. Whether high TGF-α levels have a significant impact on the biological behaviour of these tumours will be determined by clinical follow up.

### Ectopic secretion of β-HCG by bladder cancers – a clinical marker of metastasis and prognosis

R.K. Iles & T. Chard

*Department of Reproductive Physiology, St Bartholomew's Hospital Medical College, London EC1A 7BE, UK.*

Though previously considered to be a marker of the extremely rare chorioepithelioma of the bladder, immunoreactive hCG-like material can be detected in serum in ≃30% of bladder patients. It has been shown *in vitro* that seven of nine bladder tumours and four of five urothelial cell lines secrete hCG-like material into their culture media (Iles *et al.*, 1987). Immunochemical analysis showed that this material consisted almost entirely of free β-subunit (Iles & Chard, 1989). Serum and early morning urine samples from 175 patients, with various stages of bladder disease, were assayed for hCG using a β-subunit, directed RIA. Forty-six patients showed elevated levels. Elevated levels of serum immunoreactive β-hCG was detected in 16 of 21 patients (76%) with confirmed metastases, but only two of 64 patients (3%) with disease limited to the pelvis. In urine elevated β-hCG levels were detected in 14 of 57 TA/1 cases (25%), 11 of 25 T2/T4 cases (71%) and five of seven metastatic disease patients (71%) (Iles *et al.*, 1989). Immunochemical analysis of samples from this study showed that only one of seven metastatic patients and one of 21 patients with disease limited to the bladder, produced intact hCG. Since only the intact hCG α-β hetrodimer is biologically active; this explains the discrepancy between the high incidence of immunoreactive hCG expression by bladder tumours and the rarity of gonadotrophin associated clinical endocrinopathies (Iles *et al.*, 1990).

### References

ILES, OLIVER, KITAU, WALKER & CHARD (1987). *Br. J. Cancer.*, **55**, 623.

ILES & CHARD (1989). *J. Mol. Endocrinol.*, **2**, 107.

ILES, JENKINS, OLIVER, BLANDY & CHARD (1989). *Br. J. Urol.*, **64**, 241.

ILES, LEE, OLIVER, CHARD (1990). *Clin. Endocrinol.*, **32**, 355.